# Gender and thrombolysis therapy in stroke patients with incidence of dyslipidemia

**DOI:** 10.1186/s12905-018-0698-6

**Published:** 2019-01-16

**Authors:** Brice Blum, Leah Wormack, Mason Holtel, Alexandria Penwell, Shyyon Lari, Brittany Walker, Thomas I. Nathaniel

**Affiliations:** University of South Carolina, School of Medicine-Greenville, Greenville, SC 29605 USA

**Keywords:** Acute ischemic stroke, Dyslipidemia, Gender, Tissue-type plasminogen activator (rtPA), Exclusion

## Abstract

**Background:**

When untreated, dyslipidemia is a higher risk factor for stroke and stroke-related mortality in men than in women. However, when dyslipidemia is treated the risk reduction is the same, but men benefited from mortality reduction more than women. Whether there is a gender difference in exclusion criteria for the use of recombinant tissue plasminogen activator (rtPA) or thrombolysis therapy in an acute ischemic stroke subpopulation with dyslipidemia is yet to be investigated.

**Method:**

In a dyslipidemic stroke population obtained from a stroke registry, gender differences in exclusion risk factors were determined using clinical and demographic variables. Univariate analysis compared the recombinant tissue plasminogen activator (rtPA) group and the no rtPA group. Multiple regression analysis was used to determine demographic and clinical factors associated with inclusion and exclusion for rtPA in the total dyslipidemic stroke population and the subsets of the male and female population. The regression model was tested using the Hosmer-Lemeshow test, for the overall correct classification percentage. Significant interactions and multicollinearity between independent variables were examined using variance inflation factors.

**Results:**

A total of 769 patients presented with acute ischemic stroke with incidence dyslipidemia; 325 received rtPA while 444 were excluded from rtPA. Of those excluded from rtPA, 54.30% were female and 45.72% were male. In an adjusted analysis, female patients with increased age (OR = 1.024, 95% CI, 1.001–1.047, *P* < 0.05), with a history of carotid artery stenosis (OR = 7.063, 95% CI, 1.506–33.134, *P* < 0.05), and previous stroke (OR = 1.978, 95% CI, 1.136–3.442, P < 0.05) were more likely to be excluded from rtPA. Male patients with atrial fibrillation (OR = 2.053, 95% CI, 1.059–3.978, *P* = 0.033), carotid artery stenosis (OR = 2.400, 95% CI, 1.062–5.424, *P* = 0.035), and previous stroke (OR = 1.785, 95% CI, 1.063–2.998, *P* = 0.028) were more likely to be excluded from rtPA.

**Conclusion:**

Although there are some similarities in the clinical risk factors for exclusion in both male and female stroke patients with incidence of dyslipidemia, there are differences as well. Elderly female stroke patients with incidence of dyslipidemia are more likely to be excluded from rtPA, even after adjustment for the effect of confounding variables. Further research should focus on how identified clinical risk factors can be targeted and managed to improve the use of rtPA in elderly female acute ischemic stroke population with incidence of dyslipidemia.

## Background

Gender differences in clinical risk factors of acute ischemic stroke patients have been studied [1–5]. Findings indicate that women tend to develop acute ischemic strokes at an older age and have a worse prognosis when compared with men [6]. Women that develop ischemic stroke tend to have higher rates of hypertension and atrial fibrillation [7–9], while men have higher rates of smoking, dyslipidemia, coronary artery disease, and carotid artery stenosis [5, 10, 11]. The risk factors tend to increase in prevalence as the stroke population ages, resulting in a relative increase in risk for elderly female or elderly male stroke patients [12–14]. Female stroke patients are known to present with more severe symptoms, less likely to recover all functions, poor outcomes, and a general reduction in quality of life when compared to men stroke patients [15–18]. Moreover, women are more likely to present with atrial fibrillation [19], and hypertension [20], whereas men are more likely to present congestive heart disease and diabetes [21]. These studies reveal evidence of a gender difference in stroke symptoms and past clinical history.

Dyslipidemia is a major risk factor for acute ischemic stroke [22–25]. Uncontrolled levels of high-density lipoprotein (HDL-C), low-density lipoprotein (LDL-C), and total cholesterol (TC) are involved in acute ischemic stroke [26]. Dyslipidemia is known to increase the risk of stroke in males due to the expression of estrogens [27, 28]. The neuroprotective function of estrogen is seen in the younger female population; no difference was found in the risk of stroke between males and females in older populations with dyslipidemia as a risk factor [29, 30]. It is possible that age-related comorbidities often associated with elderly stroke patients [31] may complicate treatment outcomes in elderly male or female stroke patients with dyslipidemia. When untreated, dyslipidemia has a higher risk of stroke and stroke-related mortality in men compared to women [32]. When treated with statin the risk reduction is approximately equal between the genders, but men benefited more from mortality reduction than women [33, 34]. However, it is not clear whether the existing gender difference is shown in the exclusion or inclusion criteria for rtPA in a dyslipidemic stroke population.

In the general population, acute ischemic stroke has a more severe effect in women than in men when untreated [5, 8, 35]. However, treatment of acute ischemic stroke with rtPA eliminates outcome differences between genders, suggesting that women have a more robust response to thrombolytic therapy than men [36–38]. However, gender differences in a dyslipidemic subpopulation of acute ischemic stroke patients with and without rtPA is not fully understood. If a greater benefit of thrombolytic therapy continues to persist in women within the stroke population with dyslipidemia, one possibility is that there is a disproportionate distribution of clinical risk factors between male and female acute ischemic stroke with incidence dyslipidemia, such that more male or female or vice versa maybe excluded from rtPA. We tested this hypothesis in a population of female dyslipidemic acute ischemic stroke patients and compared our results with male dyslipidemic acute ischemic stroke patients. First, we characterized clinical risk factors in acute ischemic stroke patients with incident dyslipidemia that received or were excluded from rtPA. Secondly, we determined the effect of demographic and clinical risk factors that influence the exclusion or inclusion of ischemic stroke patients with incidence of dyslipidemia from rtPA. Our findings will help to identify clinical risk factors that can be targeted to eliminate gender differences and improve the use of rtPA in the management of an acute ischemic stroke population with incidence of dyslipidemia.

## Methods

### Data collection

Retrospective data was obtained from a stroke registry of patients presenting with acute ischemic stroke between January 1, 2010 and June 30, 2016 at the Greenville Health Care System (GHS). The GHS stroke registry contributes to Get with The Guidelines (GWTG)–Stroke, a national registry of the American Heart Association and American Stroke Association. The GHS registry has been described in previous studies [13, 18, 39–43]. All data including neuroimaging data were reviewed by a clinician, who determined whether the patient met the clinical case description of acute stroke. The events were categorized as ischemic stroke, transient ischemic attack, intracerebral hemorrhage, or subarachnoid hemorrhage according to descriptions of stroke from the Classification of Cerebrovascular Diseases III [44]. A stroke nurse identified and abstracted data for the patient’s demographics, clinical variables that were used for the study. Stroke risk factors were determined through evaluation of the documented patient medical history retrieved during admission to the GHS stroke unit. The data collected included demographics (age and gender), admission date, medications, clinical diagnosis, prehospital care, pre-stroke and post-stroke ambulatory status, in-hospital procedures, past medical history, and the clinical decision whether to treat the patient with rtPA or not. Documentation also included potential contraindications, patient history, characteristics of stroke presentation, physiologic status, and patient refusal of rtPA treatment. Data collection was scrutinized under quality control checks using established protocol to ascertain the quality of the data and to insure against several types of errors, including: errors in interpretation or coding, errors in data entry. This study was approved by the ethics committee of the Greenville Health system in 2017. This study is a retrospective data analysis and all data were de-identified. The study did not involve patients and no consent was required.

### Data analysis

Demographic and clinical characteristics of male and female stroke patients with incidence of dyslipidemia who were included or excluded for rtPA were analyzed using univariate analysis. Comparisons between the male and female patients were determined using a two-tailed, independent samples student t-test for the discrete numerical variables. Categorical variables were analyzed using the chi-squared test. Following the analysis of gender differences between the rtPA group and the no rtPA group, further analysis was determined using a multivariate analysis. In this analysis, a binary logistic regression was used to identify clinical factors that were associated with exclusion from the rtPA group. In the regression model, rtPA treatment was used as the dependent variable. In addition, gender was included as the primary independent variable in the regression model for the whole dyslipidemic stroke population, while the male and female sub-cohort were analyzed separately in the regression model. Although dyslipidemia was an inclusion criterion in the model, it may not appear in the final stepwise regression model if it was not significantly associated with the dependent variable in order to meet the criteria for stepwise consideration. In our analysis, the predictor variables for each logistic regression model were selected by stepwise regression and variables with *P* < 0.01 remained in the model. The binary logistic regression model was further tested by using a Hosmer-Lemeshow test. Multicollinearity and significant interactions between independent variables were examined using variance inflation factors. All statistical analysis was performed using SPSS Statistics Software Version 24.0, and a *p*-value of less than 0.05 was used to determine significance.

## Results

A total of 769 stroke patients presented with incidence of dyslipidemia (Table 1). As shown in Table 1, more females (*P* = 0.006) were excluded from rtPA when compared with male stroke patients with incidence of dyslipidemia. The characterization of stroke patients with incidence of dyslipidemia by rtPA status and gender is presented in Table 2. More females were excluded from rtPA (*P* = 0.002) when compared with male patients. Females excluded from rtPA were older (73.6 ± 12.7 vs 69.9 ± 12.7), had a higher NIH Stroke Scale rating (P = 0.002), greater mean GWTG risk of mortality (*P* = 0.046), and lower coronary artery disease (0.017) when compared with male stroke patients with incidence of dyslipidemia.

**Table 1:** Demographic and clinical characteristics of acute ischemic stroke patients with incidence of dyslipidemia.

	rtPA	No rtPA	*p*-value
Number of Patients	325	444	
Age Group: No. (%)			
<50 years	28 (8.6)	25 (5.6)	0.004*
50-59	62 (19.1)	52 (11.7)	
60-69	80 (24.6)	99 (22.3)	
70-79	75 (23.1)	129 (29.1)	
>80 years	80 (24.6)	139 (31.3)	
Mean ± SD	68.25±13.4	71.90±12.8	0.333
Gender: No. (%)			
Male	182 (56.0)	203 (45.7)	0.006*
Female	143 (44.0)	241 (54.3)	
Race: No. (%)			
Caucasian	266 (81.8)	361 (81.3)	0.920
African-American	55 (16.9)	76 (17.1)	
Other	4 (1.2)	7 (1.6)	
Medical History: No. (%)			
Hypertension	298 (91.7)	390 (87.8)	0.096
Coronary Artery Disease	124 (38.2)	183 (41.2)	0.413
Atrial Fib/Flutter	56 (17.2)	124 (27.9)	0.001*
Previous Stroke	90 (27.7)	166 (37.4)	0.005*
Previous TIA	46 (14.2)	57 (12.8)	0.594
Congestive Heart Failure	33 (10.2)	69 (15.5)	0.031*
Carotid Artery Stenosis	14 (4.3)	44 (9.9)	0.004*
Peripheral Vascular Disease	26 (8.0)	43 (9.7)	0.446
History of Smoking	93 (28.6)	83 (18.7)	0.001*
Diabetes	112 (34.5)	181 (40.8)	0.084
Medication History: No. (%)			
Antiplatelet	215 (66.2)	297 (66.9)	0.877
Antihypertension	285 (87.7)	366 (82.4)	0.054
Cholesterol Reducer	252 (77.5)	336 (75.7)	0.606
Diabetes Medication	91 (28.0)	140 (31.5)	0.302
Initial NIH Stroke Scale Group: No. (%)			
0-9	203 (62.5)	317 (71.4)	0.077
10-14	44 (13.5)	46 (10.4)	
15-20	41 (12.6)	43 (9.7)	
21-25	37 (11.4)	38 (8.6)	
Mean ± SD	9.42±6.6	7.37±6.6	0.565
Risk of Mortality GWTG Ischemic Stroke:			
Mean ± SD	5.45±6.0	4.62±5.3	0.093
Body Mass Index			
Mean ± SD	29.10±6.7	28.68±7.2	0.264

Continuous variables are represented as Mean ± S.D. and comparisons between groups were determined using Student’s T Test. Discrete variables are represented as Count (Percent Frequency) and comparisons between rtPA and non rtPA groups were made using Pearson’s Chi-Squared

**Table 2:** Demographic and clinical characteristics of acute ischemic stroke patients with incidence of dyslipidemia stratified by rtPA and gender status.

	Received rtPA			Excluded from rtPA		
	Male	Female	*p*-value	Male	Female	*p*-value
Number of Patients	182	143		203	241	
Age Group: No. (%)						
<50 years	13 (7.1)	15 (10.5)	0.002*	14 (6.9)	11 (4.6)	0.001*
50-59	46 (25.3)	16 (11.2)		23 (11.3)	29 (12.0)	
60-69	45 (24.7)	35 (24.5)		62 (30.5)	37 (15.4)	
70-79	45 (24.7)	30 (21.0)		54 (12.6)	75 (31.1)	
>80 years	33 (18.1)	47 (32.9)		50 (24.6)	89 (36.9)	
Mean ± SD	66.5±11.7	70.4±14.9	0.011*	69.9±12.7	73.6±12.7	0.002*
Race: No. (%)						
Caucasian	151 (83.0)	115 (80.4)	0.835	167 (82.3)	194 (80.5)	0.667
African-American	29 (15.9)	26 (18.2)		32 (15.8)	44 (18.3)	
Other	2 (1.1)	2 (1.4)		4 (2.0)	3 (1.2)	
Medical History: No. (%)						
Hypertension	167 (91.8)	131 (91.6)	0.961	176 (86.7)	214 (88.8)	0.501
Coronary Artery Disease	84 (46.2)	40 (28.0)	0.001*	96 (47.3)	87 (36.1)	0.017*
Dyslipidemia	74 (53.2)	63 (54.3)	0.864	41 (51.9)	26 (45.6)	0.469
Atrial Fib/Flutter	29 (15.9)	27 (18.9)	0.485	53 (26.1)	71 (29.5)	0.433
Previous Stroke	54 (29.7)	36 (25.2)	0.369	76 (37.4)	90 (37.3)	0.984
Previous TIA	31 (17.0)	15 (10.5)	0.093	31 (15.3)	26 (10.8)	0.160
Congestive Heart Failure	18 (9.9)	15 (10.5)	0.859	29 (14.3)	40 (16.6)	0.503
Carotid Artery Stenosis	12 (6.6)	2 (1.4)	0.022*	26 (12.8)	18 (7.5)	0.061
Peripheral Vascular Disease	14 (7.7)	12 (8.4)	0.818	19 (9.4)	24 (10.0)	0.832
History of Smoking	60 (33.0)	33 (23.1)	0.050*	45 (22.2)	38 (15.8)	0.085
Diabetes	64 (35.2)	48 (33.6)	0.763	76 (37.4)	105 (43.6)	0.190
Medication History: No. (%)						
Antiplatelet	129 (70.9)	86 (60.1)	0.042*	133 (65.5)	164 (68.0)	0.572
Antihypertension	157 (86.3)	128 (89.5)	0.376	163 (80.3)	203 (84.2)	0.278
Cholesterol Reducer	146 (80.2)	106 (74.1)	0.191	154 (75.9)	182 (75.5)	0.933
Diabetes Medication	48 (26.4)	43 (30.1)	0.461	60 (29.6)	80 (33.2)	0.411
Initial NIH Stroke Scale Group: No. (%)						
0-9	119 (65.4)	84 (58.7)	0.616	158 (77.8)	159 (66.0)	0.026*
10-14	24 (13.2)	20 (14.0)		19 (9.4)	27 (11.2)	
15-20	21 (11.5)	20 (14.0)		12 (5.9)	31 (12.9)	
21-25	18 (9.9)	19 (13.3)		14 (6.9)	24 (10.0)	
Mean ± SD	9.00±6.6	9.97±6.8	0.197	6.33±6.2	8.24±6.8	0.002*
Risk of Mortality GWTG Ischemic Stroke:						
Mean ± SD	5.35±5.9	5.59±6.1	0.727	4.04±4.9	5.09±5.5	0.046*
Body Mass Index						
Mean ± SD	28,6±5.8	29.7±7.6	0.153	28.0±5.7	29.2±8.3	0.080

Continuous variables are represented as Mean ± S.D. and comparisons between groups were determined using Student’s T Test. Discrete variables are represented as Count (Percent Frequency) and comparisons between rtPA and non rtPA groups were made using Pearson’s Chi-Squared

Following multivariable regression analysis of the entire population of both male and female dyslipidemic stroke patients (Table 3), six variables were associated with rtPA: history of atrial fibrillation (OR = 1.807, 95% CI, 1.140–2.862,*P* = 0.012), gender (OR = 1.675, 95% CI, 1.192–2.354,*P* = 0.003), history of previous stroke (OR = 1.847, 95% CI, 1.276–2.673,*P* = 0.001), and carotid artery stenosis (OR = 3.030, 95% CI, 1.540–5.960, P = 0.001) were significantly associated with exclusion from rtPA, while antiplatelet medication (OR = 0.539, 95% CI, 0.310–0.938,*P* = 0.029) and NIH Stroke Scale scores (OR = 0.882, 95% CI, 0.832–0.936, *P* = 0.001) were significantly associated with inclusion for rtPA in stroke patients with incidence of dyslipidemia.

**Table 3:** Factors associated with rtPA in the whole dyslipidemic stroke population.

	*B* Value	Wald	Odds Ratio	95% C.I. Lower	For OR Upper	*P* Value
Age	0.012	2.210	1.012	0.996	1.028	0.137
BMI	-0.008	0.414	0.992	0.967	1.017	0.520
NIH Stroke Scale	-0.125	17.336	0.882	0.832	0.936	<0.001*
Risk of Mortality GWTG	0.069	3.302	1.071	0.995	1.153	0.069
Gender	0.516	8.838	1.675	1.192	2.354	0.003*
Race	0.181	0.821	1.198	0.810	1.773	0.365
Atrial Fibrillation	0.592	6.350	1.807	1.140	2.862	0.012*
Coronary Artery Disease	0.136	0.520	1.145	0.792	1.655	0.471
Carotid Artery Stenosis	1.108	10.309	3.030	1.540	5.960	0.001*
Diabetes	0.380	1.442	1.462	0.787	2.716	0.230
Congestive Heart Failure	0.260	0.979	1.296	0.775	2.168	0.322
Hypertension	-0.247	0.577	0.781	0.412	1.479	0.448
Previous Stroke	0.614	10.591	1.847	1.276	2.673	0.001*
Previous TIA	-0.311	1.514	0.733	0.447	1.202	0.219
Peripheral Vascular Disease	0.062	0.043	1.064	0.590	1.921	0.836
History of Smoking	-0.337	2.308	0.714	0.462	1.103	0.129
Antiplatelet medication	-0.291	2.188	0.747	0.508	1.099	0.139
Antihypertensive medication	-0.618	4.786	0.539	0.310	0.938	0.029*
Cholesterol Reducer	-0.187	0.813	0.829	0.552	1.246	0.367
Antidiabetic medication	-0.091	0.074	0.913	0.474	1.759	0.785

Positive B values (Adj, OR > 1) denote variables more associated with rtPA exclusion while negative B values (Adj. OR < 1) denote variables more associated with rtPA inclusion. Hosmer-Lemeshow test (*P* = 0.826), Classification table (overall correctly classified percentage = 65.9%), and area under the ROC curve (AUC = 0.716) were applied to check the model fitness

A further adjusted analysis was performed with a more specific focus on the male dyslipidemic stroke population (Table 4) revealed that a history of atrial fibrillation (OR = 2.053, 95% CI,1.059–3.978, *P* = 0.033), history of carotid artery stenosis (OR = 2.400, 95% CI, 1.062–5.424, *P* = 0.035), and history of previous stroke (OR = 1.785, 95% CI, 1.063–2.998, *P* = 0.028) were significantly associated with exclusion from rtPA. Even after adjusting for confounding variables, the significant effect of NIHSS (OR = 0.879, 95% CI, 0.805–0.959, P = < 0.004) and anti-hypertensive medication (OR = 0.534, 95% CI, 0.301–0.948, *P* = 0.032) for inclusion in rtPA was still present in the male stroke patients with incidence of dyslipidemia. In the adjusted analysis for the female dyslipidemic stroke population (Table 5), NIHSS (OR = 0.887, 95% CI, 0.815–0.965, *P* = 0.005) and use of antihypertensive medication (OR = 0.347, 95% CI, 0.144–0.836, *P* = 0.018) were associated with inclusion for rtPA. Female dyslipidemic stroke patients with increased age (OR = 1.024, 95% CI, 1.001–1.047, *P* = 0.043), carotid artery stenosis (OR = 7.063, 95% CI, 1.506–33.134, *P* = 0.013), or previous stroke (OR = 1.978, 95% CI, 1.136–3.442, *P* = 0.016) were more likely to be excluded from rtPA.

**Table 4:** Factors associated with rtPA in the male dyslipidemic stroke population.

	*B* Value	Wald	Odds Ratio	95% C.I. Lower	For OR Upper	*P* Value
Age	0.005	0.142	1.005	0.981	1.029	0.707
BMI	-0.010	0.175	0.991	0.947	1.036	0.676
NIH Stroke Scale	-0.130	8.409	0.879	0.805	0.959	0.004*
Risk of Mortality GWTG	0.062	1.228	1.064	0.954	1.186	0.268
Race	0.237	0.645	1.268	0.711	2.261	0.422
Atrial Fibrillation	0.719	4.537	2.053	1.059	3.978	0.033*
Coronary Artery Disease	0.015	0.003	1.015	0.613	1.682	0.953
Carotid Artery Stenosis	0.875	4.426	2.400	1.062	5.424	0.035*
Diabetes	-0.035	0.007	0.965	0.420	2.217	0.934
Congestive Heart Failure	0.431	1.297	1.539	0.733	3.230	0.255
Hypertension	-0.339	0.528	0.712	0.285	1.778	0.467
Previous Stroke	0.579	4.798	1.785	1.063	2.998	0.028*
Previous TIA	-0.256	0.576	0.774	0.400	1.500	0.448
Peripheral Vascular Disease	0.377	0.704	1.457	0.605	3.512	0.401
History of Smoking	-0.426	1.986	0.653	0.361	1.181	0.159
Antiplatelet medication	-0.628	4.589	0.534	0.301	0.948	0.032*
Antihypertensive medication	-0.321	0.679	0.725	0.338	1.557	0.410
Cholesterol Reducer	-0.226	0.562	0.798	0.442	1.441	0.454
Antidiabetic medication	0.235	0.265	1.265	0.517	3.092	0.607

Positive B values (Adj, OR > 1) denote variables more associated with rtPA exclusion while negative B values (Adj. OR < 1) denote variables more associated with rtPA inclusion. Multicollinearity and interactions among independent variables were checked. Hosmer-Lemeshow test (*P* = 0.356), Classification table (overall correctly classified percentage = 66.7%), and area under the ROC curve (AUC = 0.726) were applied to check the model fitness

**Table 5:** Factors associated with rtPA in the female dyslipidemic stroke population.

	*B* Value	Wald	Odds Ratio	95% C.I. Lower	For OR Upper	*P* Value
Age	0.023	4.109	1.024	1.001	1.047	0.043*
BMI	-0.001	0.006	0.999	0.967	1.031	0.940
NIH Stroke Scale	-0.120	7.713	0.887	0.815	0.965	0.005*
Risk of Mortality GWTG	0.068	1.518	1.070	0.961	1.193	0.218
Race	0.108	0.147	1.115	0.640	1.940	0.701
Atrial Fibrillation	0.387	1.295	1.473	0.756	2.871	0.255
Coronary Artery Disease	0.404	1.910	1.498	0.845	2.655	0.167
Carotid Artery Stenosis	1.955	6.145	7.063	1.506	33.134	0.013*
Diabetes	1.001	3.377	2.720	0.936	7.909	0.066
Congestive Heart Failure	0.216	0.331	1.242	0.594	2.596	0.565
Hypertension	-0.049	0.010	0.952	0.369	2.456	0.919
Previous Stroke	0.682	5.820	1.978	1.136	3.442	0.016*
Previous TIA	-0.338	0.686	0.713	0.320	1.588	0.407
Peripheral Vascular Disease	-0.356	0.688	0.700	0.302	1.625	0.407
History of Smoking	-0.296	0.745	0.744	0.379	1.458	0.388
Antiplatelet medication	-0.043	0.024	0.958	0.556	1.650	0.877
Antihypertensive medication	-1.057	5.572	0.347	0.144	0.836	0.018*
Cholesterol Reducer	-0.182	0.384	0.834	0.469	1.482	0.535
Antidiabetic medication	-0.627	1.236	0.534	0.177	1.613	0.266

Positive B values (Adj, OR > 1) denote variables more associated with rtPA exclusion while negative B values (Adj. OR < 1) denote variables more associated with rtPA inclusion. Multicollinearity and interactions among independent variables were checked. Hosmer-Lemeshow test (*P* = 0.102), Classification table (overall correctly classified percentage = 66.9%), and area under the ROC curve (AUC = 0.708) were applied to check the model fitness

## Discussion

Gender differences seen within the stroke population are multifactorial with genetics, demographics, and clinical factors all playing a role. For example, stroke is greater in women than in men [9, 34], and among those with a maternal history of stroke, the risk is greater in women than men [34]. Several studies have reported evidence of a gender difference in the onset, treatment, and care of stroke [9, 34, 35]. For example, women are known to have a greater incidence of stroke than men (3% vs. 1.6%) and more severe outcomes complicated by comorbidities [36, 37]. Therefore, understanding pre-treatment demographic and clinical variables that contribute to a gender difference could help eliminate any gender treatment disparities and improve treatment outcome in stroke populations irrespective of gender.

In the univariate analysis, we found that a number of patients’ pre-treatment variables did not significantly differentiate males and females in the exclusion from rtPA. These variables include hypertension, atrial fibrillation previous stroke, dyslipidemia, previous TIA, congestive heart failure, peripheral vascular disease, carotid artery stenosis, history of smoking, diabetes, cholesterol antihypertension, antiplatelet, reduction medication, diabetes medication and body mass index. However, more females were excluded because of higher NIHSS and GWTG risk of mortality, while fewer females were excluded because of coronary artery disease compared with higher dyslipidemic male stroke patients. Moreover, male and female dyslipidemic stroke patients were significantly different in terms of age, suggesting that unadjusted gender differences in rtPA may have been masked by age.

Following adjustment for the effects of age and clinical risk factors in the whole dyslipidemic stroke population, more female dyslipidemic stroke patients did not receive rtPA. In addition, patients with carotid artery stenosis, previous stroke and atrial fibrillation were excluded rtPA. Even after the adjustment, the exclusion from rtPA was strongly associated with the female gender in the whole population. The effect of age disappeared after adjustment while the female gender was significant as an exclusion criterion for rtPA. This finding suggests that the female gender reflects differences in clinical risk factors in rtPA decision making, such that severe comorbidities play a major role in the exclusion of female dyslipidemic stroke patients from rtPA.

The effect of age and gender disappeared after the adjusted analysis in male dyslipidemic stroke patients. Men with atrial fibrillation, carotid artery stenosis, and previous stroke have the tendency of being excluded from rtPA. In the adjusted model for the female population, female patients with increased age, carotid artery stenosis, and a pre-treatment of prior stroke were more likely to be excluded from rtPA treatment. The adjusted analysis did not eliminate the effect of age in the female dyslipidemic stroke population; this suggests a pattern towards a higher exclusion of older women than men. Most of the pre-treatment variables identified in this study have been observed in other studies [1–4, 38, 39]. In our study, women were older than men that received rtPA (70.4 years vs 66.5 years), and this is similar to other studies [40, 41]. Moreover, our results indicated that women were older than men in the exclusion group (73.6 years vs 69.9 years). In our study, both male and female stroke patients with incidence of dyslipidemia were likely to be excluded because of carotid artery stenosis and prior stroke while other studies found that women were more likely to have carotid artery stenosis [42–44]. Moreover, other studies found that men tend to face the likelihood of being excluded because of previous history of stroke [45, 46]. We found that there is a tendency that both men and women may not receive rtPA because of atrial fibrillation while other studies found that women were more likely to have atrial fibrillation [42–44].

In our study, the adjusted analysis did not eliminate the effect of age or gender in the largely older female dyslipidemic stroke patients that dominated our sample population. This finding suggests that female gender in dyslipidemic stroke population maybe be directly linked to older age (70 yrs), carotid artery stenosis and previous history of stroke. It is possible that the female gender may not have the sufficient sample size power to trigger exclusion from rtPA in our younger age (70 yrs) female dyslipidemic stroke population. Many factors have been attributed to the observed effect of gender in elderly stroke. This includes the effect of cardiomyopathy in women, especially at older age [47]. The genes that control the reaction to rtPA are different in men and women [48].

In addition, the hormonal activities that promote hypercoagulability are different in men and women [45], and may affect predisposition to stroke in women. The current study took the existing observations of gender, stroke and thrombolysis therapy to a different level by assessing the effect of gender in exclusion from rtPA in a dyslipidemic stroke population. Our findings indicate that gender difference is not only related to genetic factors, but clinical risk factors and age are also markedly involved. A better management of elderly stroke patients with incidence of dyslipidemia could improve the use of rtPA and stroke treatment outcomes. Investigating therapies targeting dyslipidemia may thus be useful to improve rtPA outcomes in patients with acute ischemic stroke**.**

Limitations of this study included the following: how dyslipidemia was managed after the onset of stroke is not clear, this is a retrospective and single institution study making it difficult to extrapolate our findings to other centers. Moreover, this is a retrospective study and there is tendency for a selection bias because our data was not randomized. An important contribution of this study to existing literature in stroke neurology is our finding that gender as a risk factor for stroke with incidence of dyslipidemia is closely linked to age and clinical risk factors including carotid artery stenosis and previous stroke.

## Conclusion

The current study provides a significant addition to understanding the gender-related exclusion from rtPA associated with acute ischemic stroke with incidence of dyslipidemia. The importance of gender and age as risk factors in dyslipidemic stroke cannot be underestimated.

## Abbreviations

GWTG: Get with The Guidelines
HDL-C: High-density lipoprotein
LDL-C: low-density lipoprotein
NIHSS: National Institute of Health stroke scale
OD: Odd ratio
rtPA: Recombinant tissue plasminogen activator
TC: Total cholesterol
TIA: Trans ischemic attack
